# Preliminary Validation of the Exercise-Snacking Licensing Scale: Rewarding Exercise with Unhealthy Snack Foods and Drinks

**DOI:** 10.3390/nu10121866

**Published:** 2018-12-02

**Authors:** Jessica S. West, Kym J. Guelfi, James A. Dimmock, Ben Jackson

**Affiliations:** School of Human Sciences (Exercise and Sport Science), The University of Western Australia, 35 Stirling Highway, Crawley, Perth, WA 6009, Australia; kym.guelfi@uwa.edu.au (K.J.G.); james.dimmock@uwa.edu.au (J.A.D.); ben.jackson@uwa.edu.au (B.J.)

**Keywords:** compensatory snacking, justification, nutrition, physical activity

## Abstract

There is evidence that individuals’ compensatory health beliefs may be an important psychological driver of health behavior. Only recently, however, have researchers begun to develop and seek to validate instruments that are suited to measuring specific pairings of the diverse compensatory health beliefs that exist. The aim of this study was to provide support for key aspects of validity associated with the Exercise-Snacking Licensing Scale (ESLS), an instrument that was designed to assess individuals’ endorsement (or licensing) of unhealthy snacking behaviors around exercise. Participants (*N* = 1095) responded to a version of the ESLS that was designed to assess their licensing responses following either “light” or “tiring” physical activity, and completed additional instruments assessing dispositional, exercise-related, and diet-related constructs. Analyses indicated that scores derived from both versions of the ESLS (“light” and “tiring” physical activity) displayed a relatively consistent factor structure, favorable alpha coefficients, and meaningful correlations with variables that are theoretically aligned with licensing. Factor analytic procedures did, however, indicate that researchers may wish, in future, to consider the use (or not) of reverse-scored items within the ESLS. Together, these findings provide important insight into the structural, external, and generalizability aspects of validity for scores derived from the ESLS, and indicate that the ESLS may be a valuable instrument for the brief assessment of unhealthy licensing beliefs around exercise. Further use of the ESLS is encouraged to determine if and how these licensing beliefs actually influence subsequent snacking behaviors, and the potential downstream effects these beliefs may have in shaping health outcomes associated with exercise participation.

## 1. Introduction

Regular exercise is important for the maintenance and promotion of one’s physical health [1]. It is also widely acknowledged that consuming a healthy and balanced diet—independently and in conjunction with exercise—plays an important role in supporting health outcomes [2,3,4]. There are, however, a host of pervasive environmental and personal factors that may conflict with individuals’ long-term health and weight-management goals [5,6]. Giving in to impulses when faced with appealing and hedonically-pleasurable high-calorie foods/drinks is one such factor that can undermine weight-loss and health promotion efforts [7]. Indeed, the consumption of unhealthy, calorie-dense foods/drinks often occurs following exercise [8,9,10,11], and such behavior may, in part, explain the modest weight-loss experienced by some individuals during exercise interventions [3,12,13].

Unlike standard meal times (e.g., breakfast, lunch, dinner), which may be predominantly cued by physiological markers of hunger or time of day, unhealthy snack consumption may be particularly influenced by psychological drivers [14]. Cleobury and Tapper [14], for example, reported that the primary factor underpinning unhealthy snack-food choices (i.e., snack foods high in either fat or sugar) in overweight and obese individuals was that the food looked or smelled tempting. Interestingly, exercise has been shown—at least in some individuals—to heighten the reward value of food, including high-fat/sugar items [12]. That being the case, assessing the potential reward value of unhealthy snack foods/drinks, rather than food in general [15,16], may provide valuable insight into compensatory snacking behaviors around one’s exercise participation.

The notion (or endorsement) of unhealthy snack consumption around exercise is couched within what has been referred to as a broader “compensatory health belief” framework [17]. To illustrate, it is argued that dissonance arises when one’s desires to engage in a “negative” or indulgent behavior (e.g., to consume unhealthy snack foods/drinks) come into conflict with one’s long-term goals (e.g., weight-loss). To resolve this dissonance, individuals may attempt to justify the negative or indulgent behavior by positioning it relative to some other positive behavior that supports the long-term goal (e.g., “I can eat this unhealthy food because I have exercised today”) [17]. In that respect, individuals may consider that: (a) the indulgent behavior (e.g., unhealthy snacking) is neutralized, or compensated for, by the healthy behavior (e.g., exercise); and/or (b) that engagement in the healthy behavior (e.g., exercise) provides sufficient justification to reward oneself with, or license oneself to, an indulgent behavior (e.g., unhealthy snacking) [18].

A number of instruments have been designed to measure compensatory health beliefs for a range of behavior “pairings” [17,19], including those that exist between food and exercise [20,21]. However, these measures may not fully capture the specific “licensing” relationship that exists between exercise and subsequent unhealthy snacking behaviors. For example, compensatory health belief items around weight management, originally stemming from the Compensatory Health Beliefs Scale [17], and subsequently used by Radtke et al. [21], only include one item addressing the “food-and-exercise” pairing. In addition, although the instrument used by Poelman, Vermeer, Vyth and Steenhuis [20] contains four items assessing this pairing, these items focus on how a healthy diet/smaller portion sizes can compensate for minimal exercise (e.g., “When I eat less, it’s not necessary to have a lot of exercise”), rather than how exercise may act as justification for unhealthy eating/drinking (e.g., “because I have exercised, it’s okay to eat some chocolate cake”). 

Moshier et al. [16] addressed this limitation by developing the Compensatory Eating Motives Questionnaire (CEMQ), a self-report instrument designed to measure the different motives for compensatory eating behaviors around exercise. Scores derived from this instrument have demonstrated preliminary evidence of validity for the purpose of measuring reward, relief, and recovery motives of compensatory eating. However, the CEMQ and other food–exercise compensatory health belief instruments do not capture perceptions about the consumption of unhealthy *drinks*, which (independent of food consumption) may have important negative effects on health and weight management [22]. Also, the CEMQ focuses on food consumption in a general sense, rather than isolating *unhealthy* options. Although there is value from a weight management (and overall calorie/energy balance) perspective in understanding food–exercise compensation in a general sense, unhealthy snacks (The use of the term “snacks” is intended to broadly encompass both food *and* drinks) are often energy-dense, poor in nutritional value [15,23,24], and may be associated with negative health outcomes that cannot be compensated for by exercise, such as the management of cholesterol [4]. 

A handful of recent studies have been published in which investigators have examined compensatory (or licensing) beliefs regarding unhealthy snacking, around exercise specifically [25,26,27]. In this research, a six-item self-report instrument—which is referred to here as the Exercise–Snacking Licensing Scale (ESLS)—was used to measure individuals’ perceptions about the consumption of unhealthy snack food/drink around (and particularly following) exercise [26,27]. As well as demonstrating associations with theorized correlates (e.g., motivation [26]), scores derived from this instrument have also displayed initial evidence of validity in other ways (e.g., through acceptable internal consistency estimates) in previous studies. Nonetheless, despite the promise of the ESLS as an instrument to assess compensatory licensing beliefs relating to exercise and snack foods/drinks, a more systematic approach to validation and psychometric evaluation is needed in order to document evidence for key aspects of validity associated with scores on this instrument. Guided by established criteria for instrument validation, the aim of this study was to examine, in greater detail than has been done so previously, aspects of validity associated with scores derived from the ESLS. 

In his seminal paper, Messick [28] described six distinct aspects, or evaluative criteria, associated with construct validity (and instrument validation). Messick specifically outlined that a comprehensive understanding—and therefore, appropriate measurement—of constructs required the consideration of the following aspects of validity: content (i.e., relevance, representativeness, technical quality), substantive (i.e., theoretical rationale), structural (i.e., fidelity of the scoring structure), external (i.e., convergent and discriminant validity), generalizability (i.e., capacity to extrapolate across different populations, settings, tasks), and consequential (i.e., implications of score interpretations). Of these evaluative criteria, evidence for content and substantive aspects of validity of the ESLS has been presented previously through the integration of previous literature (during item development) and through expert review input [26]. In addition, as a result of correlations demonstrated with exercise motivation, preliminary support for the external aspect of validity has also been reported [26,27]. To date, though, relatively little support exists for the ESLS with respect to other important aspects of Messick’s framework. The aim of this study, therefore, was to present further evidence for the validity of scores derived from the ESLS within three key areas of construct validity (structural, external, and generalizability). 

The six-item ESLS contains four positively-worded, reward-focused items and two reverse-coded items, which assess beliefs around *avoiding* (rather than endorsing) unhealthy snack foods/drinks following exercise. Although reverse-scored items have some merit, such as ensuring participant vigilance and reducing acquiescence bias [29,30,31], the use of such items has become the subject of debate in recent years [30,31,32,33]. In some cases, reverse-coded items have contributed to unexpected factor structures [29,30,34] or miscomprehension [33], both of which can increase the likelihood of systematic error and pose threats to validity [34]. For that reason, it is particularly important to consider the structural aspects of the ESLS, in order to determine whether the reverse-scored items support (or impair) the quality of inferences made using the six-item instrument. With respect to external aspects of validity, it is important to test the associations between scores on the ESLS with respect to theorized exercise- (e.g., exercise motives, goals, participation) and diet-related (e.g., food attitudes, self-control, related compensatory health beliefs) correlates. Indeed, understanding in more detail the associations between ESLS scores and relevant correlates promises to contribute valuable insight into the nomological net associated with the instrument/construct.

Another aim of the study presented was to demonstrate support for the generalizability aspect of validity by considering individuals’ licensing beliefs associated with both “tiring” and “light” forms of physical activity. It has been proposed, for example, that licensing responses may be determined in part by one’s perceived mental or physical effort associated with an activity [9,10,35,36]. Therefore, it is possible that individuals might endorse licensing beliefs (regarding the consumption of unhealthy food/drinks around exercise) to differing degrees depending on the intensity of their physical activity (i.e., greater physical activity intensities may warrant greater licensing). If exercise is not strenuous or long enough to justify compensation, then the engagement of licensing beliefs may be minimized or not occur at all. This could be one explanation for why Inauen et al. [37] did not observe an increase in unhealthy snack food consumption following 5 min of exercise (i.e., aerobic step-ups). Demonstrating support for consistent structural properties (e.g., through factor analytic methods) of the ESLS, at the same time as identifying fluctuations in the magnitude of licensing beliefs at different physical activity intensities, would: (a) provide important information regarding the generalizability aspect of validity; and (b) advance our understanding of the factors involved in the activation of licensing beliefs. 

Therefore, the aim of the current study was to assess three key aspects of construct validity (structural, external, and generalizability) associated with scores derived from the ESLS. To address this aim, exploratory factor analytic procedures and internal consistency estimates both with and without reverse-coded items were conducted (structural aspect of validity), along with estimated correlations with a range of relevant constructs (external aspect of validity), and the ESLS was administered with respect to both “tiring” and “light” physical activity (generalizability aspect of validity). 

## 2. Materials and Methods

### 2.1. Participants

Male and female participants (*N* = 1095; participant characteristics are presented in the results section after data screening [details in data analysis section]) between 18 and 45 years of age and who spoke English were drawn from US, UK, or Australian populations.

### 2.2. Procedures

Recruitment and data collection occurred online via two independent recruitment panels (*SocialSci*, *Qualtrics*). Web-based data collection is now a frequently used method within the behavioral sciences [38,39,40]; online methods may reduce sample biases [39] and do not appear to yield significant differences in terms of measurement properties (i.e., factor structure) [40] when compared to traditional data collection (i.e., paper and pen) approaches. The procedures for this study were approved by the Human Research Ethics Committee at the authors’ institution. Prior to completing the survey, participants were provided with an information sheet and were asked to provide their informed consent to participate. 

For the purpose of examining the generalizability aspect of validity, the ESLS used by West et al., [26] was adapted to reflect licensing beliefs after either “tiring” or “light” physical activity. As such, 653 participants were asked to complete a version of the ESLS relating to their responses regarding tiring physical activity, and 442 completed a version with modified ESLS items that pertained to light physical activity. These two conditions were further split into two sub-samples according to the correlates that were assessed alongside licensing perceptions. Specifically, within both the “tiring” and “light” ESLS sub-samples, two different questionnaire packages that included different correlate variables were assessed. The decision to split the sub-samples in this way enabled—across the study as a whole—greater insight into external aspects of validity, and also allowed for all questionnaire packages to follow recommendations of not exceeding a completion time of 15 to 20 min [41]. 

Within both the “tiring” and “light” ESLS sub-samples, approximately half of the participants completed a survey that included “exercise-related” correlates (i.e., motivational regulation for exercise, exercise motives, and leisure time exercise behavior), whilst the other half completed a survey that was focused more toward a range of “diet-related” or “dispositional” correlates (i.e., affective and instrumental attitudes toward unhealthy snack foods/drinks, trait self-control, food-related self-control, compensatory health beliefs; see following section for more information on measures). Participants therefore received one of four possible survey packages; a “tiring” physical activity ESLS with exercise correlates (*n* = 319), a “light” physical activity ESLS with exercise correlates (*n* = 216), a “tiring” physical activity ESLS with diet-related/dispositional correlates (*n* = 334), or a “light” physical activity ESLS with diet-related/dispositional correlates (*n* = 226).

### 2.3. Measures

#### 2.3.1. Exercise–Snacking Licensing Beliefs

Licensing beliefs regarding the consumption of unhealthy food/drinks after exercise were measured using the 6-item ESLS [26,27], which was modified to relate to either “light” or “tiring” physical activity. Distinctions between light and tiring physical activity were made in the instructions to the instrument. For example, in the light physical activity condition, instructions read, “When you see the term ‘physical activity’, this refers only to light physical activity, which does not leave you feeling worn out or tired when you finish”. Definitions for “unhealthy snacks” and “unhealthy drinks” were also provided in the instructions. It was noted, for example, that “When you see the term ‘unhealthy snacks’, this refers to ‘junk’ foods that are high in fat and/or sugar, such as potato chips, hot chips/fries, chocolate, confectionary (i.e., lollies, lollipops, candy), fast food, and sugary pastries (e.g., donuts)”. To orient participants to consider their licensing-related thoughts and feelings (rather than behaviors), participants were instructed, “When responding to the statements below, please think about how you most commonly feel in the few hours immediately following a light/tiring physical activity session”. Participants rated the extent to which they endorsed licensing beliefs around consuming unhealthy snack foods/drinks after exercise on a response scale ranging from 1 (strongly disagree) to 7 (strongly agree). An example item is, “After engaging in light/tiring physical activity, I feel that I can reward myself with unhealthy drinks” (all items are provided in Table 1). Evidence of internal consistency for scores derived from the ESLS has been reported in previous work [26,27]. In this study, alpha coefficients were computed for scores derived from the six-item (light physical activity α = 0.83; tiring physical activity α = 0.85) and four-item ESLS (light physical activity α = 0.93; tiring physical activity α = 0.91). 

#### 2.3.2. Motivational Regulation for Exercise

The Behavioral Regulation in Exercise Questionnaire-2 [42] consists of 19 items designed to assess individuals’ motivational regulations for exercise. Responses are provided on a five-point scale anchored at 0 (not true for me) to 4 (very true for me). Prior to completing the 19 items, individuals were asked to consider the light/tiring physical activity in which they most commonly engage (or would engage). Example items include: “I take part in this form of light/tiring physical activity… because it’s fun” (intrinsic motivation; 4 items), “…because it’s important to me to do it regularly” (identified regulation; 4 items), “…because I feel guilty when I don’t” (introjected regulation; 3 items), “....because other people say I should” (external regulation; 4 items), and “…but I don’t see the point in doing it” (amotivation; 4 items). Subscale scores were averaged, and a relative autonomy index was subsequently calculated using weighted subscale scores [43]. Weightings were consistent with those used in previous research [44,45], whereby subscale scores were multiplied by the following weightings: amotivation −3; external regulation −2; introjected regulation −1; identified regulation +2; and intrinsic regulation +3. Higher relative autonomy index scores reflect greater autonomous, relative to controlled, exercise motivation, and are therefore considered indicators of higher “quality” motivation for exercise [44,46]. Researchers have presented support for the validity and reliability of scores derived from the Behavioral Regulation in Exercise Questionnaire-2 [26,42,44]. Internal consistency estimates (α) for scores derived from measures completed in both the light and tiring physical activity surveys in this study were as follows: amotivation (α = 0.87 and 0.93 for the “light” and “tiring” samples, respectively), external regulation (α = 0.89 and 0.91, respectively), introjected regulation (α = 0.85 and 0.81, respectively), identified regulation (α = 0.77 and 0.80, respectively), and intrinsic motivation (α = 0.94 and 0.96, respectively). In accordance with previous research [26], an inverse relationship between relative autonomy index scores and individuals’ endorsement of licensing beliefs (i.e., ESLS scores) was anticipated. 

#### 2.3.3. Exercise Motives

The Exercise Motivations Inventory-2 [47] was used to assess the goals (or potential goals) associated with individuals’ exercise participation. Specifically, although the Behavioral Regulation in Exercise Questionnaire-2 assesses *why* people exercise (e.g., for fun, because they value the outcomes of exercise, due to external pressure), the Exercise Motivations Inventory-2 is designed to assess *what* people’s motives are for exercise. The entire Exercise Motivations Inventory-2 includes 14 different exercise motives; six were selected for use in the present study. Participants were reminded to think about the most common light/tiring physical activity that they personally participated in before answering the motive statements. The exercise motives chosen measured the extent to which people exercised (or would exercise) for enjoyment (four items; e.g., “Personally, I take part in this form of physical activity… because I enjoy the feeling of exerting myself”), revitalization (three items; e.g., “…because it makes me feel good”), ill-health avoidance (three items; e.g., “…to avoid ill-health”), positive health (three items; e.g., “…to have a healthy body”), weight management (four items; e.g., “…to stay slim”) and appearance (four items; e.g., “…to help me look younger”). Items were scored on a six-point response scale ranging from 0 (not at all true for me) to 5 (very true for me). Support for the psychometric properties of scores derived from the Exercise Motivations Inventory-2 has been reported previously [16,47]. Internal consistency estimates (α) for scores derived from measures completed in both the light and tiring physical activity surveys in this study were as follows: enjoyment (α = 0.92 and 0.93 for the “light” and “tiring” samples, respectively), revitalization (α = 0.89 and 0.91, respectively), ill-health avoidance (α = 0.95 and 0.93, respectively), positive health (α = 0.96 and 0.94, respectively), weight management (α = 0.91 and 0.89, respectively), and appearance (α = 0.92 and 0.92, respectively). Negative correlations between each of these “positive” exercise motives and ESLS scores was anticipated (e.g., if an individual scored highly on exercising for weight management, enjoyment, ill-health avoidance, etc., s/he would report lower licensing beliefs). 

#### 2.3.4. Leisure Time Activity Levels

The Godin-Shephard Leisure Time Exercise Questionnaire [48] was used to assess individuals’ typical levels of mild, moderate, and strenuous physical activity. For analysis purposes, a single physical activity score was computed (using weightings applied to mild, moderate, and strenuous physical activity) to account for differences in metabolic demand of the different exercise intensities (mild multiplied by 3; moderate by 5; strenuous by 9). Scores from this measure have been identified as a reliable and valid way to assess an individual’s leisure-time physical activity levels [48,49].

#### 2.3.5. Affective and Instrumental Attitudes Toward Unhealthy Snack Foods/Drinks

Affective (i.e., enjoyment-related) and instrumental (i.e., health-related) attitudes toward specific snack foods and drinks were assessed with items adapted from West et al. [26]. Participants rated their affective and instrumental attitudes (one item each) toward chocolate, confectionery, potato chips, sweet pastries, soda, and high-fat drinks on a seven-point bipolar scale (two items total per food/drink, where 1 = not at all enjoyable/healthy and 7 = very enjoyable/healthy). Scores from these measures have been shown to display evidence of acceptable internal consistency in previous research [26]. Alpha coefficients were computed for scores derived from affective (α = 0.73 and 0.74 for the “light” and “tiring” samples, respectively) and instrumental attitudes (α = 0.89 and 0.91, respectively). A positive relationship between attitudes toward these foods/drinks and licensing (i.e., ESLS) scores was expected. 

#### 2.3.6. Self-Control

Trait self-control was assessed using the Brief Self-Control Scale [50]. This instrument contains 13 items pertaining to general self-control (e.g., “I am good at resisting temptation”), which are scored on a response scale ranging from 1 (not at all) to 5 (very much). Higher mean scores indicate a greater level of trait self-control. Scores on the Brief Self-Control Scale have previously been shown to provide reliable and valid interpretations of an individual’s trait self-control [50,51]. In this study, alpha coefficients for scores on the Brief Self-Control Scale were as follows (α = 0.85 and 0.83, for the “light” and ”tiring” samples, respectively). 

Separately, three questions specifically regarding individuals’ food-related self-control, which were constructed by Honkanen et al. [52] and based on previous studies [50], were also utilized in this study. The three items included, “I have a hard time breaking bad food habits”, “I wish I had more self-discipline when it comes to unhealthy food”, and “Sometimes I can’t stop myself from eating unhealthy food, even if I know it’s wrong”. The items were assessed using a response scale ranging from 1 (strongly disagree) to 7 (strongly agree). All questions were reverse-scored, so that higher aggregate scores indicated greater self-control. Scores from these three items have demonstrated acceptable internal consistency in recent research [26], and in this study, acceptable alpha coefficients were as follows (α = 0.89 and 0.91 for the “light” and “tiring” samples, respectively). Given that the ability to resist temptation is an important element of self-control [50], a negative relationship between self-control scores (both general and food-related) and participants’ licensing (i.e., ESLS) scores was anticipated.

#### 2.3.7. Compensatory Health Beliefs

Two distinct (but complementary) instruments to assess closely related compensatory health beliefs regarding food were included in the present study. First, in accordance with previous research [21], five items related to dieting and weight-loss derived from the original Compensatory Health Beliefs Scale [17] were used. The five items included the assessment of beliefs around food and exercise (e.g., “If one exercises one can eat without many restrictions”), as well as more general dietary behaviors (e.g., “Skipping the main dish can make up for eating dessert”). Answers were provided on a five-point response scale ranging from 0 (strongly disagree) to 4 (strongly agree), with higher scores indicating greater endorsement of compensatory health beliefs. A strength of these items is that they capture a broad range of potential compensatory behaviors (e.g., exercise, calorie restriction, use of artificial sweeteners, etc.). However, this diversity has resulted in less-than-optimal alpha coefficients for scores derived from this instrument in previous studies (e.g., α = 0.44; [21]). In the current study alpha coefficients for scores derived from this instrument were as follows (α = 0.78 and 0.76 for the “light” and “tiring” samples, respectively). 

The Diet-related Compensatory Health Beliefs Scale [20] was also used to assess diet-related beliefs specifically associated with exercise. The entire instrument includes 11 items across three separate factors. In the current study, only the “compensatory health beliefs related to exercise” subscale was used. The “portion size” and “front-of-package logo” subscales were excluded due to being deemed unnecessary. As such, participants responded to four items, including, “To maintain your weight, it is fine to have less exercise if you eat small portions”, “To maintain your weight, it is fine to have less exercise if you eat products with a front-of-package logo”, “When I eat less, it’s not necessary to have a lot of exercise”, and “When I mainly eat products with a front-of-package logo, it is not necessary to have a lot of exercise”. Ratings were provided on a five-point response scale ranging from 0 (strongly disagree) to 4 (strongly agree), where higher scores indicated stronger endorsement of compensatory health beliefs. Researchers have previously demonstrated support for aspects of validity associated with scores derived from these instruments [20], and alpha coefficients in the present study were as follows (α = 0.73 and 0.78 for the “light” and “tiring” samples, respectively). A positive relationship between ESLS scores and scores on both of these related instruments was anticipated. At the same time, given that the ESLS assesses a specific type of exercise–diet pairing (i.e., *unhealthy* snack foods/drinks)—that is not the subject of either of the other two instruments—it was anticipated that the strength of these correlations would not indicate redundancy between concepts. 

### 2.4. Data Analysis

De-identified data files from the two recruitment sites were initially collated and screened using IBM SPSS Version 25. Of the 1095 participants who met the inclusion criteria for the study, 40 individuals were excluded due to being identified as multivariate outliers when assessing individual items on the ESLS. To reduce the risk of including redundant data, an additional 35 participants were removed for having completed the questionnaire in under 4 min (it was deemed that anyone completing the survey in such a short time could not have attended properly to the questions). Therefore, data from 1020 participants were retained for analysis. To account for input errors, BMI scores outside of the range of 15–50 kg/m^2^ were deleted for 31 individuals. In addition, six scores for typical leisure time physical activity levels that were considered univariate outliers (i.e., z score > ±3.29) were also deleted to account for potential input errors or misinterpretation of the question (e.g., completing 80 strenuous exercise sessions a week is unrealistic).

The analysis of structural (and generalizability) aspects of validity began with checks of item- and aggregate-level skewness and kurtosis for both the tiring and light versions of the ESLS (see Table 1) and inter-item correlations (see Table 2). Subsequently estimated alpha coefficients were calculated to examine the internal consistency of scores derived from the tiring and light versions of the ESLS (refer to Measures section). Exploratory factor analysis (EFA) was performed on scores from the two ESLS, and factor analytic solutions were explored both with and without reverse-coded items (i.e., a six-item and four-item measure, respectively). Factor analyses were conducted using principal axis extraction, with the number of factors determined by eigenvalues [53] and investigation of the scree plot [54]. Exploratory, rather than confirmatory factor analytic methods were used, because no empirical work had previously been conducted to investigate the factor structure of the ESLS. With the generalizability aspect of validity in mind, a series of independent-samples *t*-tests were performed—using ESLS scores derived with and without the inclusion of reverse-coded items—to examine differences in the strength of participants’ licensing endorsement according to the framing of physical activity in the ESLS (i.e., tiring vs. light). Finally, having considered structural (and by splitting the measures according to tiring vs. light physical activity, also the generalizability) aspect of validity, a series of bivariate correlations were computed to examine associations between tiring/light ESLS scores and scores on all correlates related to exercise and diet. 

## 3. Results

### 3.1. Participants

Participants (*N* = 1020, males = 359, females = 661, *M* age = 30.48 ± 7.52 y, BMI = 24.87 ± 5.45 kg/m^2^) displayed a range of typical leisure-time physical activity levels (mean Godin-Shephard Leisure Time Exercise Questionnaire score across both the light and tiring physical activity conditions = 52.47 ± 45.56 arbitrary units; [48]).

### 3.2. Structural and Generalizability Aspects of Validity

Item- and aggregate-level descriptive statistics (i.e., mean, standard deviation, normality estimates) for both (i.e., “light” and “tiring”) versions of the ESLS are presented in Table 1. Item- and aggregate-level skewness and kurtosis estimates did not indicate any departures from normality for any ESLS items or aggregate scores (in either version of the survey). 

#### 3.2.1. Factor Analysis: Light Physical Activity

An EFA was performed using principal axis factoring and orthogonal varimax rotation. Initial inter-item correlations (between positively-worded items) were positive and in the desirable range [55] (see Table 2). Reverse-scored items were strongly (and positively) correlated with each other, but displayed weaker correlations with the positively-worded items. 

The Kaiser-Meyer-Olkin index was 0.67, indicating the data were sufficient for EFA. Bartlett’s test of sphericity, *χ*^2^ (15) = 2206.33, *p* < 0.001, showed that there were patterned relationships between item scores. Using an eigenvalue cut-off of 1.0, and using visual confirmation via the scree plot, there were two factors that (after rotation) explained a cumulative variance of 81.59%. Table 3 shows the factor loadings after rotation using a significant factor criterion of 0.32 [56,57]. The interpretable solution appeared to be that the four positively-worded items loaded onto factor one (internal consistency, α = 0.93), whilst the two reverse-coded items loaded separately onto factor two (α = 0.95). The diagonals of the anti-image correlation matrix for positively-worded items were all above 0.5, whereas reverse-coded items fell marginally below the acceptable range (ESLS reversed item 5 = 0.48; ESLS reversed item 6 = 0.48) [55,58]. The same analysis was repeated with reverse-coded items removed (i.e., a 4-item ESLS) and revealed a single-factor solution, as determined by the scree plot analysis and the Keiser criterion (initial eigenvalues = 3.302, 0.325, 0.272, 0.102); this solution explained a cumulative variance of 82.54% and all items loaded meaningfully onto this primary factor (factor loadings all >0.50 [55]) as shown in Table 3. Recommendations from Hair, Black, Babin, and Anderson [55] were used when interpreting the minimum factor item loadings (i.e., loadings greater than ±0.5 are considered the minimum for practical significance). 

#### 3.2.2. Factor Analysis: Tiring Physical Activity

An EFA was performed using principal axis factoring and oblique direct oblimin rotation (rather than varimax rotation as was the case in the light physical activity condition) due to the two factors correlating (*r* = 0.32) in an exploratory principal components analysis [56]. Inter-item correlations—all of which were positive—were largely consistent with those observed for the light physical activity ESLS (see Table 2). When performing the factor analyses using scores derived from the six-item ESLS, the Kaiser-Meyer-Olkin index was 0.63, indicating the data were sufficient for EFA. Bartlett’s test of sphericity, *χ*^2^ (15) = 2814.43, *p* < 0.001, showed that there were patterned relationships between the items. The diagonals of the anti-image correlation matrix were over 0.5 for both positively and negatively worded items [55,58]. Using an eigenvalue cut-off of 1.0, and using visual confirmation via the scree plot, there were two factors that explained a cumulative variance of 82.94%. The interpretable solution again appeared to be that the four positively worded items loaded on factor one (α = 0.91), whilst the two reverse-coded items again loaded separately onto a second factor (α = 0.92). Table 3 shows the pattern matrix using a significant factor criterion of 0.32 [56,57]. With reverse-coded items removed, analyses revealed evidence of a single-factor solution as determined by the scree plot analysis and the Keiser criterion (initial eigenvalues = 3.124, 0.418, 0.359, 0.100). The single-factor solution explained a cumulative variance of 78.09%, and all items loaded meaningfully onto this primary factor (factor loadings all >0.50 [55]) as shown in Table 3.

#### 3.2.3. Differences in ESLS Scores for Light Versus Tiring Physical Activity

To aid interpretations regarding the generalizability aspect of validity, all outputs described above are separated (in tables) according to the intensity of physical activity associated with the ESLS. Broadly, the consistency in terms of factor analytic findings (between the light and tiring versions of the ESLS) does appear to lend initial support for the generalizability aspect of validity. Independent-samples *t*-tests—conducted using the aggregate-level scores for the ESLS—revealed that licensing (endorsement) of unhealthy snacks around exercise was significantly stronger when participants were considering their involvement in “tiring” in comparison to “light” physical activity. Outputs from analyses using both the six-item ESLS, *t*(1018) = −2.23, *p* = 0.026, and four-item ESLS, *t*(1018) = −3.21, *p* = 0.001, revealed that participants reported greater endorsement of licensing when responding with tiring, relative to light, physical activity in mind. Mean and standard deviations for the six- and four-item ESLS, when phrased in response to light and tiring physical activity, are presented in Table 1. The absolute values (i.e., combining light and tiring physical activity conditions) of the six-item (3.39 ± 1.33) and four-item (3.37 ± 1.58) ESLS were at approximately the mid-point of the scale, indicating that there were minimal concerns regarding ceiling or floor effects for typical responses on either version of the instrument.

### 3.3. External Aspect of Validity

For the purpose of examining associations with relevant correlates, correlations using aggregate scores from both the six- (ESLS6) and four-item (ESLS4; reverse-coded items removed) instruments were computed. Correlations for both the “light” and “tiring” versions of the ESLS are presented separately (for correlation outputs in relation to exercise correlates see Table 4 and diet-related correlates see Table 5). For composite-level descriptive statistics and alpha coefficients for exercise—(Table S1) and diet-related correlates (Table S2), please refer to the Supplementary Materials.

#### 3.3.1. Exercise Correlates

With respect to exercise motivation (i.e., relative autonomy index scores computed using Behavioral Regulation of Exercise Questionnaire-2 subscales), scores on both “light” and “tiring” physical activity versions of the ESLS6 and ESLS4 displayed significant negative correlations with participants’ exercise motivation. Specifically, individuals scored lower on licensing beliefs around unhealthy snacking following exercise when they reported higher-quality (i.e., more autonomous, relative to controlled) motivation for exercise. With respect to scores on all subscales within the Exercise Motivations Inventory-2 (i.e., exercising for enjoyment, revitalization, ill-health avoidance, positive health, weight management, and appearance), significant negative correlations were observed with scores on the “tiring” ESLS6. For example, when individuals strongly endorsed exercise as a means of revitalization, for health improvement, or for weight management, they reported weaker licensing beliefs. Interestingly, the same broad pattern was observed for ESLS6 scores even when respondents were considering their responses to light physical activity (with the exception of a non-significant correlation with the weight-management motive). As indicated in Table 4, correlations between ESLS4 scores and Exercise Motivations Inventory-2 subscale scores were largely non-significant (for both “light” and “tiring” versions of the ESLS4). Finally, it was observed that ESLS6 scores were significantly and negatively correlated with participants’ leisure-time physical activity levels within the “tiring” (but not “light”) version of the ESLS (ESLS4 scores were not significantly correlated with physical activity levels). The significant correlation indicated that participants in the sample who were relatively more active were less likely to endorse the licensing of unhealthy foods/drinks following tiring physical activity.

#### 3.3.2. Diet-Related and Dispositional Correlates

Scores on both the “light” and “tiring” versions of the ESLS6 and ESLS4 were significantly correlated in a positive direction with participants’ affective and instrumental attitudes towards unhealthy snacks (see Table 5). That is, it appeared that individuals reported stronger licensing of unhealthy snacks around exercise when they rated unhealthy snacks (on the whole) as more pleasurable/enjoyable (affective attitude) and perceived them to be (relatively more) healthy (instrumental attitude). Significant negative correlations were also observed for scores on both the “light” and “tiring” versions of the ESLS6 and ESLS4, with respect to individuals’ general trait self-control and food-related self-control (i.e., participants with greater self-control reported weaker licensing beliefs). Finally, it was observed that scores on both versions of the ESLS6 and ESLS4 were significantly correlated in a positive direction with participants’ general and diet-related compensatory health beliefs. Importantly, however, the strength of these correlations (*r* range 0.22 to 0.46) indicated that ESLS scores were empirically distinguishable from scores on these existing (and related) compensatory belief instruments.

## 4. Discussion

Compensatory health beliefs represent the notion that one can counteract the effects of a negative health behavior (e.g., smoking) by engaging in a “paired” positive behavior (e.g., exercise) [17]. In some instances, these compensatory pairings may operate in the reverse direction [18,21], whereby individuals could use their engagement in a positive, healthy behavior (such as exercise) to justify, or license themselves to, an unhealthy behavior (e.g., unhealthy food). In the case of exercise and nutritional “pairings”, this form of licensing may, in part, explain the modest weight-loss experienced by some individuals during exercise-only interventions [3,12,13]. In light of the potential health implications of these beliefs, and the developing literature regarding compensatory health beliefs, there is a need for researchers to direct their attention to developing—and presenting validity evidence for—instruments that assess these forms of licensing (as well as other diverse compensatory pairings). The ESLS is a brief instrument that has been used in recent studies to assess licensing beliefs relating to the consumption of unhealthy snack foods/drinks around exercise [26,27]. Despite preliminary evidence to support the use of the ESLS [26,27], it is necessary to consider in more detail several key aspects of validity associated with scores derived from this instrument. With that in mind, the aim of this investigation was to assess the structural, external and generalizability aspects of validity associated with scores on the ESLS among a large representative sample. Analyses revealed support for the utility, refinement, and continued use of the ESLS; in the material that follows, broad conclusions that can be drawn from the present study are identified, and important recommendations for future research are presented.

Analyses regarding the structural aspect of validity for scores on the ESLS indicated that researchers may wish to use the instrument in the future with or without the reverse-coded items. Analyses for the six-item (i.e., original, previously used) ESLS indicated that the reverse-coded items may load onto a second (separate) factor. It is not uncommon for reverse-coded items to load separately from positively-worded items [29,30,34], and researchers may conclude that the benefits of including reverse-coded items (e.g., maintaining participant alertness, minimizing acquiescence bias) outweigh any modelling or interpretive complications brought about by the second factor. Alternatively, researchers may justify the use of a single-factor (six-item) ESLS on the basis that the reverse-coded (second) factor may simply be a methodological artefact and not conceptually meaningful in its own right [29,59]. It is possible, of course, that the second factor may be substantively important, and may have emerged due to a substantive underlying reason. For example, there is a well-established approach-avoidance distinction in the psychological literature [60], reflecting people’s tendencies to focus on either approaching a desired state or avoiding an undesired state. Rather than simply being a product of methodology (i.e., wording), therefore, it is possible that the positively-worded (“allowing reward”) ESLS items may represent an approach notion, whereas the negatively-worded (“avoiding unhealthy snacks”) items may represent an altogether different underlying (i.e., avoidance) motive. If this is the case, it may be beneficial for researchers to not only consider a two-factor approach to scoring the ESLS, but also to investigate whether a more comprehensive assessment of the avoidance motive may be warranted (e.g., by adding additional items to the reverse-coded section of the ESLS so as to improve conceptual coverage). 

A practical alternative to using the six-item ESLS—which was supported empirically in this investigation—would be for researchers to move forward with a four-item version of the instrument (i.e., without reverse-coded items). Indeed, balanced against the documented benefits of reverse-coded items, it has been noted recently in the psychometric literature [31] that reverse-coded items may contribute to unexpected factor structures [29,30,34] and miscomprehension [33]. In support of this approach, it is important to highlight that the reverse-coded items displayed somewhat inconsistent inter-item correlations (when compared to those observed between positively-worded items). The validation of psychological instruments is an iterative process, and given that work on the ESLS is at an early stage, it would likely be premature to present here a “preferred” strategy out of those discussed above (e.g., a four-item ESLS, a six-item ESLS with or without acknowledgement of the potential second factor). What is important to note, however, is that there appears sufficient support for either strategy. For both the four- and six-item ESLS, it was observed that items and composite scores followed a relatively normal distribution; analyses also revealed support for the internal consistency of scores from both the four- and six-item measures. That being the case, when drawing conclusions about the optimal approach—at least with respect to the structural aspect of validity for the ESLS—it is encouraged that researchers take into account their specific research questions and intended modelling strategies. 

It was noteworthy that scores on the four- and six-item ESLS displayed relatively consistent associations with correlates (perhaps with the notable exception of Exercise Motivations Inventory-2 subscale scores; see Table 4). That is, it appears that any decision about the inclusion of reverse-coded items may not, on the whole, have a substantial effect on the external aspect of validity for ESLS scores. It was encouraging that ESLS scores aligned with dispositional, diet-related, and exercise correlates, such that individuals were more likely to endorse licensing beliefs when (for example) they: (a) reported lower-quality (i.e., more controlled) motivation for exercise; (b) had stronger affective and instrumental attitudes toward unhealthy foods/drinks; (c) were (relatively) low on general and food-related self-control and (d) tended to more strongly endorse other related compensatory health beliefs. These findings were consistent for scores derived from both the four- and six-item instrument, and were also present for “light” and “tiring” versions of the ESLS. Taken together, the inspection of correlations within this study provide important support for the external aspect of validity, and also demonstrate that individuals’ licensing perceptions regarding unhealthy snacks around one’s exercise involvement—when assessed using the ESLS—are empirically distinguishable from other compensatory health beliefs.

It is important to highlight that analyses associated with the external aspect of validity in this study may aid in identifying modifiable correlates that represent suitable targets for interventions designed to minimize unhealthy licensing beliefs (and potentially, practices). With respect to exercise motivation, these findings corroborate those reported by West et al. [26], further supporting the robustness of the ESLS within different populations, and indicate that interventions designed to encourage more positive exercise experiences (e.g., characterized by enjoyment and interest; see [11,61]) may have downstream benefits in terms of alleviating licensing beliefs post-exercise. Similarly, in terms of dispositional and dietary factors, it was interesting to observe a negative relationship between self-control and licensing even when individuals were considering light intensity bouts of physical activity (for both the four- and six-item composite score). There are examples in the literature of training programs that have been used to successfully bolster individuals’ self-control “stores” [62], and it appears from the results presented in this study that the benefits of such programs could possibly extend to the restriction of unhealthy licensing beliefs around exercise participation. However, further research is required to confirm this proposition. Finally, those who seek to promote health improvement and weight loss through physical activity programs may benefit from considering the positive correlations observed between attitudinal constructs and ESLS scores (for four- and six-item composite scores on both light and tiring versions of the survey). Changing long-held affective attitudes (e.g., that chocolate and high-fat drinks are enjoyable) through training response inhibition to unhealthy food stimuli, may provide one possible avenue for future interventions [63]. Alternatively, one’s instrumental attitudes may be susceptible to modification through the provision of compelling information and educational material [64,65]. The results presented in this study indicate that such approaches may prove to be effective for dampening the unhealthy licensing response around exercise. Indeed, although intervention-based work has begun to emerge surrounding the modification of exercise-specific licensing beliefs [27], future efforts in this area may be strengthened by drawing from the correlations observed in this study.

One noteworthy strength of this validation study is that the results provided support for the use of different versions of the ESLS. More specifically, it was demonstrated that the findings regarding factor structure, internal consistency, normality, and correlations (both inter-item and with other constructs) were largely replicable regardless of whether individuals were responding to the ESLS in terms of light or tiring physical activity involvement. As well as offering important evidence for the generalizability aspect of validity, these findings provide a platform for the use of the ESLS across a myriad of modalities and intensities of physical activity in the future. In addition, whilst displaying consistency in terms of these aspects of validity, it was interesting that the absolute magnitude (or strength) of ESLS scores *did* differ significantly for the light vs. tiring versions of the instrument. That is, individuals tended to more strongly endorse unhealthy licensing responses when considering their involvement in tiring (compared with light intensity) physical activity. This observation was noteworthy insofar as it provided further evidence for the validity of the ESLS—specifically, it demonstrated that ESLS scores were sensitive to physical-activity intensity, supporting the role of perceived effort in compensatory behaviors [9,10,35]. Meanwhile, from a practical perspective, the stronger licensing responses associated with “tiring” physical activity may be important for practitioners to bear in mind when considering the potential side-effects they should guard against when prescribing or supervising high-intensity physical activity.

### Limitations and Future Directions

The process of construct validation occurs through the accumulation of numerous studies and the refinement of instruments based on empirical evidence [66]. This study provides an important platform for further work in this area, as it represents the first systematic approach to the assessment of structural, external and generalizability aspects of validity for the ESLS [28]. The conclusions drawn from this study should, however, be considered in light of its limitations. First, it is important to note that *actual* snacking or dietary behaviors was not assessed. In order to generate a more comprehensive understanding of the behavioral implications of licensing beliefs, and to further widen the nomological net surrounding the ESLS, researchers are encouraged to examine food and drink intake (a) in naturalistic surroundings through the use of food [67,68] and snack food [69,70] diaries, and (b) through laboratory-based protocols that offer the benefit of controlled methods to quantify healthy and unhealthy food/drink intake following exercise [11]. In a similar manner, although a broad range of correlates were examined, indicators of health status were not assessed. As a result, this investigation cannot make any conclusions about the potential health outcomes associated with licensing perceptions of this kind. For future research, it would be valuable to examine the degree to which licensing beliefs—assessed using the ESLS—relate to, and potentially influence, downstream health outcomes (e.g., weight, blood pressure, cholesterol).

Although the findings presented in this study advance our understanding of the validity of scores derived from the ESLS, there is also value in future research that employs test-retest procedures to document stability and/or change for the four- and six-item ESLS [55]. Repeated assessments may provide valuable information regarding intra-individual variability in licensing responses, which is important so as to inform future intervention efforts. Finally, it is worth noting that participants in this investigation were responding to a “hypothetical” exercise scenario, and were not rating licensing perceptions in advance of, or following, an actual bout of exercise. In the future, researchers may wish to assess licensing beliefs directly before or after an exercise session [26], and examine if and how licensing responses fluctuate in response to physical activity of different modes, duration, and intensity.

## 5. Conclusions

In conclusion, these findings provide support for aspects of structural, external and generalizability validity associated with scores derived from the ESLS, and in turn, suggests that a four- or six-item ESLS may be an effective tool for practitioners and researchers seeking to measure and modify individuals’ licensing responses around exercise and unhealthy snacking. From a conceptual perspective, this study offers valuable insight into key aspects of validity for different versions of the ESLS, and broadens what is known about the correlates associated with individuals’ licensing beliefs relating to unhealthy snack intake around exercise. Having a brief and effective tool to assess licensing beliefs around exercise and unhealthy snacking may: (a) highlight individuals that are at high risk of undermining their long-term health and wellbeing goals when embarking on an exercise program, and; (b) provide insights into how exercise interventions can be delivered, to minimize the risk of compensatory snacking around exercise (e.g., through increasing autonomous forms of motivation for exercise). A crucial next step in the use of the ESLS—and instruments assessing other compensatory health beliefs—is to begin to understand if, when, and how these beliefs may play a role in shaping one’s dietary behavior and influence health outcomes associated with physical-activity participation. 

## Figures and Tables

**Table 1:** Item- and aggregate-level descriptive statistics, and skewness and kurtosis estimates for “light” (*n* = 410) and “tiring” (*n* = 610) physical activity versions of the Exercise–Snacking Licensing Scale (ESLS).

Item/Variable	Int.	M	SD	Skew/Kurt
1. I feel that I can reward myself with unhealthy drinks	L	3.09	1.82	0.52/−0.82
	T	3.36	1.82	0.31/−0.93
2. I feel that I can allow myself to consume unhealthy snacks	L	3.38	1.75	0.27/−0.87
	T	3.69	1.70	0.02/−0.90
3. I feel that I can allow myself to consume unhealthy drinks	L	3.16	1.73	0.42/−0.74
	T	3.43	1.73	0.24/−0.90
4. I think I can have unhealthy snacks because I’ve earned them	L	3.10	1.77	0.48/−0.74
	T	3.54	1.78	0.09/−1.1
5. I focus on avoiding unhealthy snacks (R)	L	4.48	1.79	−0.30/−0.88
	T	4.57	1.74	−0.32/−0.80
6. I focus on avoiding unhealthy drinks (R)	L	4.60	1.84	−0.35/−0.90
	T	4.67	1.85	−0.39/−0.90
7. ESLS 6 item aggregate variable	L	3.28	1.31	0.14/−0.29
	T	3.46	1.34	0.00/−0.41
8. ESLS 4 item aggregate variable	L	3.18	1.61	0.44/−0.50
	T	3.50	1.55	0.14/−0.67

Note: these final sample sizes are adjusted (from those identified in the procedures section) due to screening procedures described within the data analysis section. Int. denotes the intensity level of the ESLS, where L = light physical activity, and T = tiring physical activity. R = Indicates items that were subsequently reverse-coded. Skew/Kurt = skewness and kurtosis estimates.

**Table 2:** Correlation matrix between ESLS items for light (*n* = 410) and tiring (*n* = 610) physical activity versions of the instrument.

Item	Int.	2	3	4	5	6
1. I feel that I can reward myself with unhealthy drinks	L	0.69 **	0.79 **	0.76 **	0.15 **	0.21 **
	T	0.63 **	0.78 **	0.68 **	0.22 **	0.30 **
2. I feel that I can allow myself to consume unhealthy snacks	L	--	0.86 **	0.78 **	0.14 **	0.11 *
	T	--	0.81 **	0.74 **	0.33 **	0.28 **
3. I feel that I can allow myself to consume unhealthy drinks	L		--	0.73 **	0.16 **	0.22 **
	T		--	0.61 **	0.26 **	0.35 **
4. I think I can have unhealthy snacks because I’ve earned them	L			--	0.11 *	0.09
	T			--	0.26 **	0.24 **
5. I focus on avoiding unhealthy snacks (R)	L				--	0.91 **
	T				--	0.85 **
6. I focus on avoiding unhealthy drinks (R)	L					--
	T					--

Note: correlations rounded to two decimal places. ** *p* < 0.01, * *p* < 0.05. ESLS = Exercise-Snacking Licensing Scale. Int. denotes the intensity level of questionnaires where L = light physical activity and T = tiring physical activity. R = Indicates reversed item (reversed prior to the correlations shown).

**Table 3:** Factor loadings, eigenvalues, and alpha coefficients for six- and four-item versions of the light (*n* = 410; varimax rotation; rotated factor matrix loadings) and tiring (*n* = 610; direct oblimin rotation; pattern matrix loadings) physical activity versions of the ESLS.

Item	Int.	6-Item ESLS		4-Item ESLS
		F1	F2	F1
ESLS item 1	L	0.83		0.84
	T	0.82		0.82
ESLS item 2	L	0.89		0.89
	T	0.87		0.87
ESLS item 3	L	0.91		0.92
	T	0.88		0.89
ESLS item 4	L	0.86		0.85
	T	0.79		0.79
ESLS rev item 5	L		0.91	Removed
	T		0.93	Removed
ESLS rev item 6	L		0.99	Removed
	T		0.92	Removed
Initial Eigenvalues	L	3.42	1.79	3.30
	T	3.50	1.48	3.12
% of variance	L	56.98	29.89	82.54
	T	58.33	24.61	78.09
α	L	0.93	0.95	0.93
	T	0.91	0.92	0.91

ESLS = Exercise-Snacking Licensing Scale. Int. denotes the intensity level of questionnaires where L = light physical activity and T = tiring physical activity. F1 and F2 denote Factor 1 and Factor 2.

**Table 4:** Correlations with exercise correlates for four- and six-item versions of “light” (*n* = 199) and “tiring” (*n* = 297) ESLS.

Variable	Int.	2	3	4	5	6	7	8	9	10
1. Licensing (6-item ESLS score)	L	0.90 **	−0.34 **	−0.23 **	−0.19 **	−0.21 **	−0.22 **	−0.04	−0.15 *	−0.11
	T	0.92 **	−0.30 **	−0.14 *	−0.16 **	−0.24 **	−0.27 **	−0.14 *	−0.18 **	−0.13 *
2. Licensing (4-item ESLS score)	L	--	−0.28 **	−0.03	0.01	−0.03	−0.05	0.13	0.04	−0.09
	T	--	−0.33 **	−0.01	−0.03	−0.11	−0.14 *	0.02	−0.02	−0.08
3. Motivation for exercise	L		--	0.64 **	0.60 **	0.37 **	0.47 **	0.07	0.17 *	0.17 *
	T		--	0.59 **	0.58 **	0.28 **	0.38 **	0.10	0.16 **	0.19 **
4. Exercising for enjoyment	L			--	0.89 **	0.62 **	0.71 **	0.38 **	0.51 **	0.22 **
	T			--	0.91 **	0.54 **	0.61 **	0.37 **	0.51 **	0.26 **
5. Exercising for revitalization	L				--	0.65 **	0.73 **	0.40 **	0.56 **	0.21 **
	T				--	0.59 **	0.66 **	0.39 **	0.55 **	0.30 **
6. Exercising to avoid ill-health	L					--	0.87 **	0.59 **	0.66 **	0.08
	T					--	0.84 **	0.59 **	0.64 **	0.25 **
7. Exercise for positive health	L						--	0.57 **	0.68 **	0.14
	T						--	0.60 **	0.72 **	0.23 **
8. Exercise for weight management	L							--	0.77 **	0.04
	T							--	0.71 **	0.10
9. Exercising for appearance	L								--	0.02
	T								--	0.17 **
10. Godin-Shephard leisure time exercise score	L (*n* = 179)									--
	T (*n* = 262)									--

Note: ESLS = Exercise-Snacking Licensing Scale. Int. denotes the intensity level of questionnaires where L = light physical activity and T = tiring physical activity. ** *p* < 0.01, * *p* < 0.05. Motivation for exercise = measured by the relative autonomy index of the Behavioral Regulation of Exercise Questionnaire-2. Exercising for: enjoyment, revitalization, to avoid ill-health, for positive health, weight management or appearance, measured by subscales of the Exercise Motivations Inventory-2.

**Table 5:** Correlations with diet-related and dispositional correlates for four- and six-item versions of “light” (*n* = 211) and “tiring” (*n* = 313) ESLS.

Variable	Int.	2	3	4	5	6	7	8
1. Licensing (6-item ESLS score)	L	0.90 **	0.26 **	0.18 **	−0.23 **	−0.20 **	0.33 **	0.30 **
	T	0.91 **	0.28 **	0.27 **	−0.19 **	−0.20 **	0.38 **	0.22 **
2. Licensing (4-item ESLS score)	L	--	0.31 **	0.21 **	−0.21 **	−0.24 **	0.43 **	0.39 **
	T	--	0.27 **	0.30 **	−0.17 **	−0.22 **	0.46 **	0.30 **
3. Affective attitudes	L		--	0.05	−0.15 *	−0.30 **	0.16 *	0.15 *
	T		--	0.14 *	−0.25 **	−0.26 **	0.10	0.02
4. Instrumental attitudes	L			--	0.06	0.07	0.33 **	0.21 **
	T			--	0.03	0.02	0.41 **	0.32 **
5. Trait self-control	L				--	0.65 **	−0.17 *	−0.29 **
	T				--	0.60 **	−0.14 *	−0.10
6. Food-related self-control	L					--	−0.20 **	−0.34 **
	T					--	−0.13 *	−0.05
7. Compensatory health beliefs	L						--	0.71 **
	T						--	0.69 **
8. Diet-related compensatory health beliefs	L							--
	T							--

Note: ESLS = Exercise-Snacking Licensing Scale. Int. denotes the intensity level of questionnaires where L = light physical activity and T = tiring physical activity. ** *p* < 0.01, * *p* < 0.05.

## Supplementary Materials

The following are available online at http://www.mdpi.com/2072-6643/10/12/1866/s1, Table S1: Composite-level descriptive statistics and alpha coefficients within exercise correlates in the light (*n* = 199) and tiring (*n* = 297) physical activity conditions, Table S2: Composite-level descriptive statistics and alpha coefficients within diet-related and dispositional correlates in the light (*n* = 211) and tiring (*n* = 313) physical activity conditions.
