# Supplementary material for: Preliminary Validation of the Exercise-Snacking Licensing Scale: Rewarding Exercise with Unhealthy Snack Foods and Drinks
Source: Nutrients. 2018 Dec 2;10(12):1866. doi: 10.3390/nu10121866 (PMC6316787; doi:10.3390/nu10121866)
Supplement: Supplementary file 1 [file nutrients-10-01866-s001.pdf]

Supplementary Table S1. Composite-level descriptive statistics and alpha coefficients within exercise correlates in the light (*n* = 199) and tiring (*n* = 297) physical activity conditions

Variable	Intensity	Mean	SD	Skew / Kurt	α
1. Motivation for exercise	L	8.85	6.50	- .42 / - .62	---
	T	6.70	7.34	- .35 / - .48	---
2. Exercising for enjoyment	L	3.39	1.31	- .67 / - .29	0.92
	T	3.20	1.48	- .65 / - .67	0.93
3. Exercising for revitalisation	L	3.42	1.27	- .74 / .03	0.89
	T	3.27	1.45	- .81 / - .27	0.91
4. Exercising to avoid ill-health	L	3.36	1.43	- .73 / - .24	0.95
	T	3.55	1.33	- .98 / .39	0.93
5. Exercise for positive health	L	3.67	1.34	- 1.13 / .66	0.96
	T	3.87	1.20	- 1.27 / 1.51	0.94
6. Exercise for weight management	L	2.97	1.51	- .49 / - .74	0.91
	T	3.37	1.42	- .85 / - .10	0.89
7. Exercising for appearance	L	2.99	1.49	- .49 / - .58	0.92
	T	3.23	1.39	- .73 / - .24	0.92
8. Godin-Shephard leisure time exercise score	L (*n* = 179)	51.73	48.61	3.04 / 13.85	--
	T (*n* = 262)	53.01	42.36	2.11 / 6.43	--

Note: Intensity denotes the intensity level of exercise where L = light physical activity and T = tiring physical activity. Motivation for exercise = measured by the relative autonomy index of the Behavioural Regulation of Exercise Questionnaire- 2, where higher scores denote greater autonomous (relative to controlled) motivation towards exercise. Exercising for: enjoyment, revitalisation, to avoid ill-health, for positive health, weight management or appearance, measured by subscales of the Exercise Motivations Inventory -2, where higher scores denote greater agreeance to exercising for stated reason

Supplementary Table S2. Composite-level descriptive statistics and alpha coefficients within diet-related and dispositional correlates in the light (n = 211) and tiring (n = 313) physical activity conditions

Variable	Intensity	Mean	SD	Skew / Kurt	α
1. Affective attitudes	L	4.94	1.12	-.25 / - .21	0.73
	T	4.93	1.17	-.60 / .45	0.74
2. Instrumental attitudes	L	2.26	1.08	1.18 / .88	0.89
	T	2.38	1.23	1.48 / 2.00	0.91
3. Trait self-control	L	3.06	.72	.22 / - .18	0.85
	T	3.12	.69	.11 / - .17	0.83
4. Food-related self-control	L	3.62	1.73	.31 / - .83	0.89
	T	3.57	1.71	.31 / - .73	0.91
5. Compensatory health beliefs	L	1.02	.82	.89 / .71	0.78
	T	1.02	.79	.92 / .64	0.76
6. Diet-related compensatory health beliefs	L	1.20	.85	.79 / 1.15	0.73
	T	1.12	.84	.77 / .29	0.78

Note: Intensity denotes the intensity level of exercise where L = light physical activity and T = tiring physical activity.
